# Profiling of Naturally Occurring Antibodies to the Thomsen-Friedenreich Antigen in Health and Cancer: The Diversity and Clinical Potential

**DOI:** 10.1155/2020/9747040

**Published:** 2020-03-23

**Authors:** Oleg Kurtenkov

**Affiliations:** Department of Virology and Immunology, National Institute for Health Development, Hiiu 42, Tallinn 11619, Estonia

## Abstract

The Thomsen-Friedenreich (TF) antigen is expressed in a majority of human tumors due to aberrant glycosylation in cancer cells. There is strong evidence that humoral immune response to TF represents an effective mechanism for the elimination of cancer cells that express TF-positive glycoconjugates. The presence of naturally occurring antibodies to tumor-associated TF and cancer-specific changes in their levels, isotype distribution and interrelation, avidity, and glycosylation profile make these Abs a convenient and ubiquitous marker for cancer diagnostics and prognostics. In this review, we attempt to summarize the latest data on the potential of TF-specific Abs for cancer diagnostics and prognostics.

## 1. Introduction

Altered glycosylation is a characteristic feature of cancer cells, which is closely associated with tumor progression and metastasis [1–4]. Glycans are involved in fundamental cancer-related processes, such as cell signalling and communication, tumor cell dissociation and invasion, and cell-matrix interactions, as well as angiogenesis and metastasis formation [3, 5–7]. The expression or unmasking of the so-called Thomsen-Friedenreich cryptantigen (TF, CD176) on red blood cells after exposure to bacterial neuraminidases was first described in 1927 by Thomsen [8] and further specified by Friedenreich [9]. But only the fundamental studies by the G. Springer group established that TF is actually an oncofetal pancarcinoma antigen that is expressed in a majority of carcinomas [10].

The tumor-associated carbohydrate antigens (TACAs) or glycans (TAGs), including the Thomsen-Friedenreich antigen, and related glycopeptide epitopes may be autoimmunogenic and recognized by autoantibodies [11–14]. A broad spectrum of natural and adaptive anti-glycan Abs is present in human serum in health and disease, showing a rather stable level over time in healthy people [15–24]. There is strong evidence that a majority of them is a result of the innate and/or adaptive immune response to microbial carbohydrates [25–27]. An advantage of autoantibodies (AAbs) to tumor-associated antigens (TAAs) as biomarkers over tumor-derived products is their production in large quantities, especially in the early stages of cancer, and long half-life due to the limited proteolysis and clearance. However, only 10-30% of cancer patients exhibit a specific humoral immune response to a single tumor-associated antigen although the combination of several AAb markers may appreciably improve the accuracy of diagnostics [28–31]. At the same time, the inherent antigenic heterogeneity of tumors makes the use of adaptive AAbs to TAAs as cancer biomarkers very problematic, especially if a single target is used.

In contrast to the adaptive AAbs to TAAs that appear during tumorigenesis, a decreased level of naturally occurring TF-specific Abs (TF Abs) in cancer patients has been known since the pioneered studies of the G. Springer group in the 1980s [15], but no further special in-depth analysis of this phenomenon has been done possibly because of the focus being shifted to the role of cell-mediated immunity (CMI) in tumor immunosurveillance. Further drawbacks in the understanding of this decrease were not seriously analysed, partly because of the fact that these naturally occurring Abs are normally present in health, their level is rather stable over time, and no individual retrospective data regarding the TF Ab level before tumor development are usually available. It is to be noted that no special conditions that are associated with the increase of natural TF Abs, including autoimmunity and infections, have been described. Thus, although changes in the individual level of TF Abs are fairly difficult to interpret, a very low Ab level may be very suspicious for cancer.

Interestingly, up to date, there is no clear understanding of the pathophysiological role of natural TF Abs in health and disease though it is difficult to imagine that the rather high levels of these Abs of various isotypes would not have any important role for the host. Clinically observed prognostic improvements in cancer patients with a high level of TF-specific IgG Abs [17, 32–34] and encouraging experiments with TF-specific monoclonal antibodies (MAbs) [35, 36] indicate that the TF-specific innate and/or adaptive immune response is an important part of cancer immunosurveillance, and the TF antigen is a promising molecular target for cancer immunotherapy [14, 37–40]. This short review focuses mostly on the clinical potential of natural TF-specific Abs in cancer diagnostics and prognosis.

## 2. The Thomsen-Friedenreich Antigen

Aberrant cell surface glycosylation is often observed to take place in cancer cells, being involved in cancer cell adhesion, signaling, and invasion [1–3, 41–43]. The Thomsen-Friedenreich antigen (Gal*β*1-3GalNAc *α*-O-Ser/Thr) (TF, CD176) is a mucin-type O-linked disaccharide which is overexpressed in tumor cells and is associated with tumor progression, invasive growth, and high metastatic potential, which suggests its important function in cancer cell survival [10, 14, 42, 44].

The oncofetal origin of TF makes it mildly antigenic, allowing it to be perceived as “self”-like by the immune system. It is found in a cryptic form in many membrane glycoconjugates, sialic acid being one of the most important molecules for masking TF in normal tissues by terminal sialylation. The TF glycotope becomes exposed during tumorigenesis as a tumor-associated glycan (TAG) [10, 32, 37, 45]. Approximately 70-90% of carcinomas, including breast, stomach, colon, bladder, and prostate, carry TF on the tumor cell surface though the percentage of positive cases varies among diferent carcinoma types. The presence of TF during an early fetal phase, its absence in noncarcinomatous postfetal tissues, and the association with carcinoma suggest that TF is a stage-specific oncofetal antigen [46]. In tumor tissues, TF is expressed on many glycoproteins, such as mucin Muc1, CD44v6, CD133, and integrins [42, 47–49].

The aberrant overexpression of mucins in cancer cells and their altered glycosylation may lead to different, quite opposite effects on tumor immunity, from the expression of cancer-specific immunodominant glycoepitopes (TF, Tn [GalNAca1*α*-], sTn [NeuNAc-*α*2-6GalNAc *α*1-]) to the reduction of T cell effector functions [50]. TF is expressed on the surface of human leukemic cells on apoptosis-associated glycoproteins, such as CD95 and DR4, and TF Abs activate apoptotic pathways and induce apoptosis of TF-positive cells [51]. Thus, TF could be a promising target for an apoptotic therapy of cancer. The expression of TF was found to be associated with a distinct molecular subtype of gastric cancer and may be used as a new marker of microsatellite instability [52]. Interestingly, an increased TF as well as disialo-TF antigen structure was recently observed in O-linked glycan preparations of IgA1 from patients with breast cancer [53]. A high proportion of cancer cells coexpresses TF and CD44 as a marker of cancer-initiating or stem cells [54, 55]. A majority of carrier molecules for TF are actually cancer stem cell markers that differ from their normal counterparts in expression of TF, and they could be promising targets for tumor therapies [47, 54].

The TF is expressed by some enteric bacteria such as *E. coli* O86, *Bacteroides xylanisolvens*, and *Helicobacter pylori* [25, 56–58]. The TF exposure on a red blood cell (RBC) often occurs in infections with neuraminidase-producing microbes such as pneumococci, *Escherichia coli*, *S. pneumoniae*, *Bacteroides*, and influenza virus [59, 60], inducing the hemolytic-uremic syndrome [60]. There is evidence that hemolysis observed in infants with *Clostridium perfringens* infection after donor plasma transfusion may be due to RBC desialylation [61]. However, it remains unclear whether these syndromes are associated with any changes in the level of TF Abs in the circulation. It is suggested that the expression of TF in aged red blood cells (on desialylated glycophorins) may be one of the signals triggering the elimination of senescent red cells via the natural TF antibody-dependent mechanism [62], which could be a possible way to induce an adaptive humoral TF-specific immune response in normal individuals.

Several mechanisms have been proposed to explain the increased TF occurrence in cancer, such as alteration of the relative activities of glycosyltransferases that are responsible for the biosynthesis of complex O-glycans, the enhanced availability of the nucleotide sugar substrate UDP-galactose for the core 1 *β*1,3 Gal-transferase (the only enzyme which forms core 1 from the Tn antigen precursor), the acidification status of Golgi apparatus in cancer, and changes in the cellular Cosms (Core 1 beta1,3-Gal-T-specific molecular chaperone) expression [42]. The metabolic changes in and around tumor cells due to hypoxia and an enhanced pH in tumor cell cytoplasm may be also involved [5, 49, 63].

It is to be noted that in contrast to the TF expression via the desialylation of O-glycans, the increased sialylation of many glycoproteins and gangliosides is also one of the main characteristics of malignant transformation [7, 64]. In addition, the increased level of free and conjugated forms of sialic acids appears to be a frequent phenomenon in cancer, for instance, the increased *α*2-3-linked sialylation of prostate-specific antigen in prostate cancer [65], the increased serum glycoprotein sialylation in multiple myeloma [66], or an increase in disialobiantennary N-linked glycans on serum IgA1 in breast cancer [53]. However, the mechanisms behind the sialylation-related changes in different cancers remain poorly understood, and in many cancers, these changes show the lack of cancer specificity.

Thus, the TF oncofetal glycan structure represents a pancarcinoma tumor marker of exceptional specificity, being expressed among normal tissues only in the human placenta in the first and second trimesters of pregnancy [46, 67]. The cell-surface presentation of TF epitopes makes them an “ideal” candidate for targeting since they are both specific and therapeutically accessible. However, the low immunogenicity of glycans needs new approaches to improve it, for instance, by using the combined glycopeptide antigens, mimetics, TF-positive microbiota, or the manipulation on the glycosylation pattern of TF Abs [7, 68–71].

## 3. TF-Specific Antibodies in Health and Nonmalignant Conditions

A pool of naturally occurring anti-glycan Abs (produced by B1 cells) that belongs to the IgM, IgG, and IgA isotypes is present in human blood in normal healthy individuals. Natural Abs (NAbs) react with microbial antigens (in cooperation with lectins prebound to the microbe), certain alter-self components, immune complexes, apoptotic cells, or danger-associated molecular patterns (DAMPs), such as asialylated glycoproteins with exposed terminal galactose residues [11, 22, 23, 27]. Screening for anti-glycan Ab reactivity patterns showed the prevalence of IgM natural Abs that are germline-encoded and not affinity-matured [11, 22, 23, 72]. This is also true for TFalpha-specific IgM antibodies whose concentration in serum is about ten times higher than that of TF IgG [73]. In contrast, the adaptive IgMs appear after an immunological challenge, and their level normally falls during the development of the IgG response [24]. Aberrant Ab responses to carbohydrate Ags have been observed in certain autoimmune diseases, such as systemic lupus erythematosus and systemic sclerosis, and may play a pathogenetic role [23, 74–77]. The pioneered studies by the G. Springer group applied the neuraminidase-treated blood group O red blood cells that expressed the TF disaccharide on glycophorins and the hemagglutination technique [44, 78]. It was shown that all individuals demonstrated rather high agglutination titers. The application of synthetic TF-polyacrylamide- (PAA-) conjugates [16, 17, 22, 73, 79] revealed a broad spectrum of anti-carbohydrate antibodies in healthy individuals. Naturally occurring TF antibodies of different isotypes were present in the circulation in health and disease, and the IgM TF Abs usually dominated. The affinity-purified TF-specific IgGs reacted with both Gal beta13GalNAc-alpha (TFalpha) and TFbeta anomers [18, 80], and Ab responses to both anomers were found to be similar. The IC50 values for TF-PAA conjugates with TF Abs typically ranged in various sera from 2 to 5 × 10^−8^ M [18]. The strong binding of affinity-purified TF Abs to both TF*α* and TF*ββ* anomers was demonstrated, but the binding to TF*ββ* was twice higher, and the TF*ββ*-PAA conjugate was a more potent inhibitor of antibody binding [18]. In healthy individuals, the level of TF Abs varied substantially among individuals but remained rather stable at the individual level [81, 82], being thus rather conserved, as has been shown for many natural self-reactive antibody levels [11].

One of the reasons for interindividual variations in the level of TF antibodies may be the association of their level with the ABH and Lewis blood group phenotypes. It has been shown that the serum level of anti-glycan Abs, including those of non-ABH blood group-related glycans, is correlated with blood group phenotype [16, 81, 83]. When using a fully synthetic TF-hapten-polyacrylamide conjugate as an antigen, we found that blood donors of Le(a-b+)/secretor type showed the highest anti-TF IgM level regardless of the ABO(H) blood group [16]. Conversely, the related group of gastric cancer patients revealed the most pronounced decrease in IgM TF Abs. This was also true for IgG Abs. It is tempting to speculate that blood group phenotype influences the TF antigen-positive intestinal flora which is thought to be an antigenic stimulus for natural TF Ab synthesis and may differ among individuals of various Le phenotypes, thus modulating the anti-carbohydrate Ab profile and antitumor resistance mechanisms.

There is strong evidence that TF Abs may be induced by TF-positive enteric bacteria [25, 56–58]. The increase of the IgM TF Ab level after per os *Bacteroides xylanisolvens* and ovatus D-6 TF-positive strain application [56, 84] supports the idea that the microbiome induces the generation of systemic Ag-specific Abs against sugar epitopes, including TF. An increased IgG response to TF has been found in *H. pylori*-infected individuals, but mostly in those with Lewis (a-) phenotype [85]. This systemic impact of *H. pylori* infection may be explained by the TF epitope expression in *H. pylori* as was demonstrated when using several TF-specific monoclonal antibodies [58]. Unlike IgG antibodies, the IgM response was not closely related to *H. pylori* serology and a dramatic decrease of the TF IgM level was found especially in Le (a-b+)-type patients with gastric cancer.

Interestingly, the proportion of *H. pylori*-infected individuals was also related to the ABH and Lewis blood group phenotype, being significantly higher in Le (a+b-) compared to other Lewis phenotypes [86]. It is known that the TF epitope is expressed on type 3 mucin-type chains of nonsecretors [87] but is further fucosylated in secretors [88]. The differences in the binding of TF-specific 9H8 MAbs between NCTC 11637 *H. pylori* strain and clinical isolates of *H. pylori* [58] suggest that the TF epitope expression varies appreciably between the strains. This might be explained by polymorphism in the glycosylation patterns of gastric glycoconjugates, which is related to the ABH and Lewis phenotype of the host and associated with the density of *H. pylori* colonization or the degree of TF expression in a given *H. pylori* strain. This TF expression polymorphism may also be related to variations in *H. pylo*ri infection association with gastric cancer development.

We reported recently that the total IgG samples purified on PtG sorbents contained a lot of so-called “hidden” TF IgG Abs [89]. The inhibition of such a hidden reactivity by addition of IgG-depleted serum was indicative of the important role of serum-derived factors such as TF-positive or cross-reactive ligands that remained in the IgG-depleted serum and reacted with anti-TF IgG Abs again and masked the latter making them HAbs. Such a phenomenon was absent in the case of natural anti-*α*Gal epitope (Gal*α*1-3Gal) Abs that were used as a distinctive control for other anti-glycan naturally occurring Abs, suggesting that the purification of IgG per se was not the reason for HAb appearance. These data show that the free serum TF Abs in the circulation are only the “tip of the iceberg” and that the serological testing with the total serum does not reflect the whole picture, and the HAb analysis could tell us more about other players, including tumor-specific targets.

Thus, the appearance of TF Abs already at an early age after birth, their presence in all individuals, and the ability to interact with tumor-associated TF glycotopes make them a good candidate for modulation of natural antitumor mechanisms, especially in risk groups, as well as for treatment of cancer with immunotherapy (see Section 4).

## 4. TF-Specific Abs and Cancer

As mentioned above, a broad spectrum of serum anti-glycan Abs of all Ig isotypes are present in the circulation in health and disease, including cancer. The G. Springer group was the first to note that TF-specific Abs decreased in breast cancer patients and also in individuals with precancerous breast lesions [90]. Moreover, the low level of these Abs was shown to be associated with a high risk for cancer development in patients with premalignant breast lesions [44]. In addition, an increase in the anti-T Ab level was found after mastectomy for carcinoma [91]. These findings have been further confirmed by many other studies using various TF glycoconjugates as antigen in ELISA and other methods.

A significant decrease of IgM and a much lower drop in TF IgG Abs were detected in gastric cancer patients irrespective of the disease stage [16, 17, 92]. Patients with breast cancer also revealed similar variations at all stages of the disease [33]. However, the level of TF IgA Abs was higher in cancer patients. In contrast, in the serum of patients with colon cancer, a lower level of TF IgG was observed only at the early stages of disease [93]. In gastric cancer patients, a strong correlation was observed to exist between anti-TF IgG and anti-MUC1 IgG antibody levels (*P* = 0.0001) [17], which speaks about the possibility that these Abs are directed to the TF glycopeptide epitope on MUC1 mucin. The ratio of IgG/IgM and IgG/IgA in colon cancer patients was significantly lower compared with controls [93], and similar data were obtained for breast cancer patients, including those with the early stages of the disease in both cancer types [92]. Сompared to healthy controls, the decrease of TF IgG in patients with gastric cancer (estimated as a proportion of weak responders) was less pronounced than that of IgM weak responders, thus indicating that the IgG/IgM ratio was lower in gastric cancer, especially in Le b+/secretor phenotype individuals [16]. These data show that the decrease of naturally occurring TF Abs in serum is a common cancer-related phenomenon, and IgM, which is known to be a dominant natural antibody isotype, is more informative in this respect.

The proportion of “hidden” TF IgG Abs (see Section 3) was lower in cancer patients [89], possibly due to the elimination of TF IgG after its interaction with tumor-derived TF-positive carriers because part of Abs can be masked by these ligands in immune complexes and remain undetectable by conventional ELISA. Moreover, these HAbs were sialylated higher, and the SNA lectin-reactive TF Abs of patients with cancer exhibited a lower avidity [89, 94]. The relatively high level of TF-specific IgG HAbs, especially in cancer patients, suggests that TF Abs play an important role in the elimination of TF-positive material from the circulation.

It remains yet unclear whether there is a link between the degree of TF expression in the tumor and the level of anti-TF antibodies in the circulation or the decrease of TF Ab level. We believe that the reduction of TF antibodies in cancer patients may represent in part a secondary phenomenon and be due to the expression of TF in the tumor or the appearance of TF-positive glycoconjugates (MUC1, etc.) in the circulation and elimination of TF antibodies via formation of immune complexes. However, based on this logic, it is difficult to explain why the decrease of the Ab level takes place already in the early stages of cancer.

Another reason for the antibody level decrease may be that the low level of natural TF antibodies is actually inherent to a given host, which reflects its blood group phenotype and microbiota profile. We have shown that blood group A gastric cancer patients revealed the strongest suppression of the anti-TF Ab level irrespective of age, disease stage, or tumor morphology [81]. We also reported on the decrease of TF IgG mostly in advanced cancer, whereas the TF IgM level was decreased irrespective of the stage, thus reflecting the preexisting low level of TF Abs [16, 17, 33]. If TF antibodies contribute to antitumor resistance, it is tempting to assume that their low level is a risk factor for cancer, as was shown by the G. Springer group during a long-term follow-up of patients with premalignant breast conditions [32, 44]. At the same time, the decrease of TF IgG in cancer implies that no adaptive immune response is actually present in patients with cancer.

It seems that there is some kind of enrichment of weak TF IgM responders among individuals who are predisposed to gastric cancer. We suggest that the efficacy of natural TF Ab-mediated antitumor reactivity depends mostly on Ab-mediated reactions in the circulation, thus protecting patients against distant metastasis. Instead, in situ (in tumor tissue), the common effect of antitumor-associated immune effector cells and TF Abs may act as antitumor or tumor-promoting forces depending on the tumor microenvironment.

Many attempts have been made to induce TF-specific immunity in mice and man [36, 95, 96]. The TF Abs generated by immunization of mice with T/Tn (9 : 1) purified from blood group O erythrocytes with different adjuvants showed a specific complement-dependent cytotoxicity and protected against the T/Tn-positive mammary adenocarcinoma cell challenge [96]. It has been shown that only TFalpha-specific MAbs and not TFbeta-specific MAbs inhibit the proliferation of epithelial tumor cells [97]. The humanized TFalpha anomer-specific JAA-F11 MAbs have demonstrated a very high specificity to TFalpha, produced an Ab-dependent cellular cytotoxicity in TF-positive breast and lung tumor cell lines, and suppressed the in vivo tumor progression in a human breast cancer xenograft model in SCID mice [36]. However, some of the tested human and mouse MAbs to TF exhibited proliferative effects on human colon cancer cell lines which express TF [98].

There is strong evidence that the risk of specific cancers is associated with alterations of human microbiome, pointing to a possible contribution of the immune response to commensal microbiota to the risk of some cancer types [99]. The microbiome inducing induction of antigen-specific antibodies against microbial sugar epitopes, including TF, was proposed as a possible way to modulate antitumor immunity through dietary supplementation of selected commensal bacteria [84].

We have tested the hypothesis that HP infection that affects the majority of mankind may alter the natural immune response to TF antigen, thus modulating natural immune mechanisms against cancer. It was shown for the first time that the TF epitope is expressed in surface membrane glycoconjugates of *H. pylori* [58] and associated with an increased immune response to TF in infected individuals [85]. A better survival rate was found in *H. pylori*-seropositive patients compared with seronegative individuals. Moreover, the survival of patients with early gastric cancer (stage 1) was dramatically better (*P* < 0.00001) in *H. pylori*-infected patients with a higher level of TF-specific IgG antibodies (strong responders) than in weak responders [58]. The recent meta-analysis provided further evidence that the *H. pylori* infection is an indicator of good prognosis in European gastric cancer patients [100]. Notably, a higher proportion of IgG strong TF responders was found in *H. pylori*-infected gastric cancer patients irrespective of the stage of cancer. This suggests that the changes observed should not be considered as a secondary tumor-induced event.

Notably, the eradication of *H. pylori* infection led to a decrease of IgG TF antibodies [85], which further speaks in favour of the important role of microbiota in the induction of TF-specific humoral immune response. These findings imply that *H. pylori* may be indirectly involved in gastric carcinogenesis via modulation (upregulation) of natural cancer-related immune mechanisms and further support the idea that TF Abs may play an important role in tumor immunosurveillance.

## 5. Antibody Glycosylation Profiling in Health and Cancer

Immunoglobulins (Igs) are glycosylated molecules, and by now, it is clear that the N-glycans of the Fc-fragment strongly influence IgG-Fc*γ* receptor interactions and thus the Fc-mediated effector mechanisms [101, 102]. Several studies have demonstrated that NAbs usually exhibit a sialylated pattern which confers an anti-inflammatory nature, in contrast to Ag-specific adaptive Abs that are either agalactosylated or asialylated and exhibit proinflammatory properties [103–105]. Compared to healthy individuals, there is a marked change of serum Ig glycosylation in individuals with autoimmune diseases, infections, and tumors [106–113]. Most studies have been performed using the total IgG preparations from the peripheral blood. Unlike cancer, an increase of the G0F (agalactosylated, asialylated, and fucosylated) IgG glycoform is the most prominent change in a variety of chronic inflammatory and autoimmune diseases, thus promoting a proinflammatory state [76, 103, 108, 114, 115]. The appearance of IgG AAbs, especially their agalactosylated glycoforms, can predate the development of autoimmune disease symptoms by many years whereas the presence of naturally occurring IgM autoantibodies, which are usually sialylated higher, might provide some protection [116]. However, less is known about the glycosylation patterns of total serum IgM, obviously due to limitations in technology and the complexity of IgM glycans.

The serum IgG glycosylation profiling has shown cancer-specific changes in multiple cancer types [53, 64, 109, 110, 113, 117] and a diagnostic and prognostic potential in various malignancies [107, 113, 118, 119]. The distinct tIgG profile (a higher agalactosylation and a lower sialylation ratio of IgG1) has shown a high diagnostic accuracy (>90%) at discrimination of patients with autoimmune pancreatitis and pancreatic cancer [113]. A similar diagnostic potential was found for the aberrant N-glycan score of Igs for patients with urothelial carcinomas [120]. The increased level of the *α*2,6-linked sialylation of IgA1 in breast cancer has been reported to be a significant predictor of distant metastases [53]. However, although changes in the structure of total IgG glycans are associated with various diseases, the possible role of Fc glycans in tumor immunity is not yet fully understood.

The profiling of 32 different N-glycans of total serum IgG by using the liquid chromatography-electrospray ionization-mass spectrometry (LC-ESI-MS) method revealed a significant increase of agalactosylated IgG glycoforms (GnGnF, GnGn(bi)F) and a decrease of galactosylated (AGn(bi), AGn(bi)F, AA(bi), and AAF) and monosialylated IgG glycoforms (NaAF, NaA(bi)) in patients with gastric cancer. To our knowledge, we have provided the first evidence that the changes in Fc glycan profile may predict the survival of patients with gastric cancer. A higher level of fully sialylated glycans and an elevated expression of glycans with bisecting GlcNAc were associated with a better survival rate [119].

However, it is important to note that the total serum IgG glycosylation profile may significantly differ from that of antigen-specific IgG Abs involved in the pathogenesis of a specific disease [108, 121–123], suggesting the presence of disease-specific IgG changes of potential clinical importance. A shift towards agalactosylated IgG glycoforms was more prominent among HIV-specific antibodies (gp120) compared to total serum IgG [124]. It is notable that the IgG glycosylation profile can be tuned via vaccination in an antigen-specific manner [125]. It has been shown that changes of Fc-glycosylation were more profoundly altered at the site of the disease where more dramatic differences were observed, for instance, in the synovium during rheumatoid arthritis disease [126] and in the cerebrospinal fluid in multiple sclerosis [127]. Although it is well established that antibodies are very heterogeneous by glycosylation and functionally, very limited data are available on the glycodiversity of Abs to tumor-associated antigens, and of the currently used cancer biomarkers, only a few studies have been reported on the analysis of TAG-specific Ab glycosylation [89, 128–132].

In contrast to total IgG, the TF-specific IgG detected in the purified total IgG preparation from the serum of gastric cancer patients showed a higher level of galactose-specific ConA lectin binding that may also be considered as a sign of IgG hyposialylation, which was associated with a worse survival rate in these patients [128]. Interestingly, no such a difference was found for other natural IgG antibodies to a xenogenic alpha-Gal glycotope [128]. In fact, a very low level of sialylation (SNA binding) was observed in these IgG preparations [131]. Notably, this shift in SNA reactivity was even more pronounced compared with the benign group (chronic gastric diseases). These changes demonstrated a good sensitivity and specificity for stomach cancer, ranging up to 80% [129]. In contrast, in serum samples, the sialylation of TF Abs showed a very high increase (*P* < 0.0001) in cancer patients irrespective of the disease stage, gender, or tumor morphology [129]. The SNA index (SNA binding/Ab level ratio) was equally higher for TF IgG, IF IgM, and TF IgA. The SNA-positive TF Abs (a pool of all isotypes) revealed a significantly higher avidity only in cancer patients compared with both healthy controls and the benign group [94]. The high SNA binding/TF IgM level ratio was associated with poor prognosis whereas the higher avidity of SNA-reactive TF Abs was associated with a benefit in survival of stage 3 cancer patients. Some discrepancies between purified total IgG preparations and the serum IgG may be explained by the presence of so-called hidden Abs that are present in the total IgG preparations but remain undetectable in serum and may be otherwise glycosylated in health and cancer [89], thus distorting the real picture. The low level of ConA and fucose-specific AAL lectin binding to TF IgG was associated with a survival benefit in cancer patients, especially in those with stages 3-4 of the disease [131]. Interestingly, the galactosylation (ConA reactivity) of other natural Abs (antiаlpha-Gаl IgGs) was not changed in patients with gastric cancer [128]. In addition, there is no correlation between the ConA reactivities of TF and *α*Gal IgG nor between the ConA reactivity of either of them and total IgG galactosylation, suggesting an independent character of their changes. This indicates that the evaluation of the total IgG glycosylation profile does not reflect the pattern of glycosylation of antigen-specific IgGs. This also implies that the glycosylation pattern of Abs against the target antigens involved in the pathogenesis of a specific disease may be more informative than just the level of IgG Abs to a specific antigen. Notably, changes in the anti-TF ConA reactivity were more pronounced at the early stages of cancer, suggesting that these changes are not induced by tumor growth *per se* but rather precede tumor development. The higher level of TF IgG ConA reactivity (galactosylation) was associated with a lower survival rate of patients with cancer.

Recently, we established the increased *α*2,6 sialylation of TF-specific Abs (a pool of all TF Ab isotypes) also in patients with colon [130] and breast cancers [92]. Moreover, some changes showed a good diagnostic potential and association with patient long-term survival. For instance, patients with a high level of TF IgM and a low SNA binding to TF antibodies demonstrated a very high specificity for gastric cancer with an ACC value equal to 100% and a worse long-term survival rate during a follow-up period from 40 to 150 months (*P* < 0.004), especially in patients with intestinal-type tumors [129].

Glycan-protein interactions serve as the most common means of microbial adhesion, as well as tumor cell colonization, by TAG interaction with carbohydrate-binding receptors (lectins) [133–136]. Alteration of cancer cell glycans interferes with several key molecular processes, leading to tumor progression and poor prognosis [3, 6, 42]. The expression of cancer-associated TF on MUC1 and its interaction with galectin-3 promote cancer cell adhesion to endothelium, encouraging thus directly cancer metastasis [133]. This could explain the fact that the expression of TF Ags in tumor cells is associated with poor prognosis [10, 32, 45, 126, 137].

MUC1 expression is increased in many epithelial cancers, which is associated with a high metastatic potential and poor prognosis [136, 138, 139] whereas the presence of sialylated MUC1 glycoform was shown to be associated with a better prognosis in patients with breast cancer [140, 141]. The concentration of galectin-3 is also increased up to fivefold in the sera of cancer patients [142, 143], including those with the early stages of cancer. The interaction of the circulating Gal-3 with cancer cells expressing TF promotes metastasis [134, 136]. The inhibition of the adhesion of circulating TF-positive tumor cells or micrometastasis to the endothelium via the galectin-3 pathway has been proposed as a mechanism for the antimetastatic action of TF-specific Abs [134], including JAA-F11 MAb, which is highly specific for alpha-anomeric TF [35, 39]. A similar effect has been demonstrated for a single-chain TF Ab variable fragment [144].

Amazingly, no data about the effect of natural TF Abs (or their glycosubsets) on tumor cell-galectin-endothelium interactions have been presented yet though this could provide a possible route for the design of gal-3 inhibitors with improved selectivity. We speculate that the TF antigen on the circulating cancer cell may be blocked by natural TF Abs, thus competing with Gal3 binding and protecting against cancer cell adhesion to endothelium and metastasis. Another possible approach is the use of TF antigen mimetics to interfere with the Gal-3-mediated cancer cell adhesion and metastasis [145].

A general conclusion that can be made from the above findings is that TF Abs reveal cancer-specific changes in their level and glycosylation. Encouraging metastasis inhibition experiments by TF-specific MAbs clearly indicate that TF could be an important target for passive and active immunotherapies in TF-expressing tumors [35, 40]. The glycodiversity of Abs is now a topic of interest because of a possibility to construct Ab glycoforms with the predicted potential [71, 121, 146, 147], thus improving cancer immunotherapy potential.

Unfortunately, no data about the TF MAb glycosylation profile have been presented so far, which could explain some controversies in their effect on tumor cells, including the enhancement of tumor cell proliferation [148]. One of the possibilities is a more thorough characterization of TF antibodies, including their anomeric specificity [149]. Since a cryptic form of TF is present on many self and microbial glycoconjugates, it could be expected that many mechanisms involved in glycosylation machinery should influence the TF expression, thus modulating the level of natural TF-specific Abs as well as their glycosylation and functional properties.

## 6. Conclusions

The ubiquitous presence of naturally occurring Abs to tumor-associated TF antigen makes them a unique mechanism for tumor immunosurveillance. The TF-specific Ab level and profile and several host-dependent factors, such as blood group phenotype and microbiota-related mechanisms, may alter tumor-host immunological interplay and influence the clinical outcome. A consistent cancer-related decrease of TF-specific Abs and their increased sialylation are rather cancer-specific phenomena of clinical importance that may be considered as an integral indicator of tumor-host interplay and could serve as biomarkers for cancer diagnosis and prognosis. Hence, these Abs and their sialylation deserve further study.

There is still no convincing evidence that changes in the TF Ab level are associated with autoimmunity or other pathologies possibly due to the fact that, except cancer, the TF expression is a very rare phenomenon and that the immunogenicity of glycans is low. The latter could also explain a relatively modest effect of vaccination with TF-conjugates in cancer immunotherapy [95]. The use of glycopeptide epitopes, including microbial glycoconjugates and mimetics, seems to be more encouraging due to their higher specificity for cancer and the ability to overcome the low immunogenicity of carbohydrates. Commensal bacterial strains that carry TFalpha structures may be appropriate candidates for tumor vaccines [57]. The presence of naturally occurring TF Abs in every individual suggests their safe immunotherapeutic application to cancer patients.

There is an urgent need to further specify how such factors as host microbiome, age, gender, blood group phenotype, the presence of hidden TF Abs, the impact of nonmalignant conditions, and cancer type may affect the clinical value of TF Ab testing and whether the consideration of these factors could improve cancer diagnostics and prognostics. The profiling of TF Ab glycodiversity could be a promising approach in this respect. Unfortunately, there are still no data about TF antibody glycodiversity after the vaccination with various TF-positive conjugates that could be a means to improve the IT efficacy and explain as well as avoid some controversies in their effect on tumor cells, including the possibility to enhance tumor cell proliferation [148].

New possibilities for Ab modification, such as the modulation of the glycosylation status of TF Abs by targeting specific glycosyl transferases, may foster the identification of novel therapeutic strategies in cancer. There is growing evidence to suggest that the inclusion of additional characteristics such as Ab glycosylation pattern profiling, isotype interrelationships, and avidity could appreciably improve the diagnostic and predictive value of the approach. Future efforts should focus on the definition of specific glycosylation of naturally occurring Abs to TF and other cancer-related glycans to select Ab subsets that could specifically modulate the antitumor immune response and antimetastatic potential of TF-specific antibodies in a given host. Further retrospective and prospective analysis of TF-specific Ab signatures is needed to assess their efficacy in clinical settings.

## References

[1] Hakomori S. (1989). Aberrant glycosylation in tumors and tumor-associated carbohydrate antigens. *Advances in Cancer Research*.

[2] Varki A., Kannagi R., Toole B. P., Varki A., Cummings R. D., Esko J. D. (2009). Glycosylation changes in cancer. *Essentials of Glycobiology*.

[3] Pinho S. S., Reis C. A. (2015). Glycosylation in cancer: mechanisms and clinical implications. *Nature Reviews Cancer*.

[4] Munkley J., Elliott D. J. (2016). Hallmarks of glycosylation in cancer. *Oncotarget*.

[5] Häuselmann I., Borsig L. (2014). Altered tumor-cell glycosylation promotes metastasis. *Frontiers in Oncology*.

[6] Rodrigues J. G., Balmaña M., Macedo J. A. (2018). Glycosylation in cancer: selected roles in tumour progression, immune modulation and metastasis. *Cellular Immunology*.

[7] Pearce O. M. (2018). Cancer glycan epitopes: biosynthesis, structure and function. *Glycobiology*.

[8] Thomsen O. Z. (1927). Ein vermehrungsfahiges Agents als veranderedes isoagglutinatorischen verhaltens der roten Blutkorperchen, eine bisher unbekannte Quelle der Fehlbestimmungen. *Zeitschrift für Immunitets Forschungen*.

[9] Friedenreich V. (1930). Production of a specific receptor quality in red cell corpuscules by bacterial activity. *The Thomsen Haemagglutination Phenomenon*.

[10] Springer G. F. (1984). T and Tn, general carcinoma autoantigens. *Science*.

[11] Vollmers H. P., Brändlein S. (2007). Natural antibodies and cancer. *Journal of Autoimmunity*.

[12] Kobold S., Lütkens T., Cao Y., Bokemeyer C., Atanackovic D. (2010). Autoantibodies against tumor-related antigens: incidence and biologic significance. *Human Immunology*.

[13] Wandall H. H., Blixt O., Tarp M. A. (2010). Cancer biomarkers defined by autoantibody signatures to aberrant O-glycopeptide epitopes. *Cancer Research*.

[14] Monzavi-Karbassi B., Pashov A., Kieber-Emmons T. (2013). Tumor-associated glycans and immune surveillance. *Vaccines*.

[15] Springer G. F., Desai P. R., Scanlon E. F. (1976). Blood group MN precursors as human breast carcinoma-associated antigens and "naturally" occurring human cytotoxins against them. *Cancer*.

[16] Kurtenkov O. (1999). Natural IgM and IgG antibodies to Thomsen-Friedenreich (T) antigen in serum of patients with gastric cancer and blood donors: relation to lewis (a,b) histo-blood group phenotype. *Acta Oncologica*.

[17] Kurtenkov O., Klaamas K., Mensdorff-Pouilly S., Miljukhina L., Shljapnikova L., Chuzmarov V. (2007). Humoral immune response to MUC1 and to the Thomsen-Friedenreich (TF) glycotope in patients with gastric cancer: relation to survival. *Acta Oncologica*.

[18] Smorodin E. P., Kurtenkov O. A., Sergeyev B. L., Pazynina G. V., Bovin N. V. (2004). Specificity of human anti-carbohydrate IgG antibodies as probed with polyacrylamide-based glycoconjugates. *Glycoconjugate Journal*.

[19] Smorodin E. P., Sergeyev B. L., Kurtenkov O. A. (2014). The characterization of IgG antibodies to GalNAc beta-terminated glycans of gastric cancer survivors. *Experimental Oncology*.

[20] Huflejt M. E., Vuskovic M., Vasiliu D. (2009). Anti-carbohydrate antibodies of normal sera: findings, surprises and challenges. *Molecular Immunology*.

[21] Schwartz-Albiez R. (2012). Naturally occurring antibodies directed against carbohydrate tumor antigens. *Advances in Experimental Medicine and Biology*.

[22] Bovin N., Obukhova P., Shilova N. (2012). Repertoire of human natural anti-glycan immunoglobulins. Do we have auto-antibodies?. *Biochimica et Biophysica Acta*.

[23] Bovin N. V. (2013). Natural antibodies to glycans. *Biochemistry*.

[24] Díaz-Zaragoza M., Hernández-Ávila R., Viedma-Rodríguez R., Arenas-Aranda D., Ostoa-Saloma P. (2015). Natural and adaptive IgM antibodies in the recognition of tumor-associated antigens of breast cancer (Review). *Oncology Reports*.

[25] Springer G. F., Tegtmeyer H. (1981). Origin of anti-Thomsen-Friedenreich (T) and Tn agglutinins in man and in white Leghorn chicks. *British Journal of Haematology*.

[26] Galili U., Mandrell R. E., Hamadeh R. M., Shohet S. B., Griffiss J. M. (1988). Interaction between human natural anti-alpha-galactosyl immunoglobulin G and bacteria of the human flora. *Infection and Immunity*.

[27] Khasbiullina N. R., Bovin N. V. (2015). Hypotheses of the origin of natural antibodies: a glycobiologist's opinion. *Biochemistry*.

[28] Casiano C. A., Mediavilla-Varela M., Tan E. M. (2006). Tumor-associated antigen arrays for the serological diagnosis of cancer. *Molecular & Cellular Proteomics*.

[29] Nolen B. M., Lokshin A. E. (2010). Autoantibodies for cancer detection: still cause for excitement?. *Cancer Biomark*.

[30] Heo C.-K., Bahk Y. Y., Cho E.-W. (2012). Tumor-associated autoantibodies as diagnostic and prognostic biomarkers. *BMB Reports*.

[31] Lacombe J., Mangé A., Solassol J. (2014). Use of autoantibodies to detect the onset of breast cancer. *Journal of Immunology Research*.

[32] Springer G. F. (1997). Immunoreactive T and Tn epitopes in cancer diagnosis, prognosis, and immunotherapy. *Journal of Molecular Medicine*.

[33] Kurtenkov O., Klaamas K., Rittenhouse-Olson K. (2005). IgG immune response to tumorassociated carbohydrate antigens (TF, Tn, *α*Gal) in patients with breast cancer: impact of neoadjuvant chemotherapy and relation to the survival. *Experimental Oncology*.

[34] Smorodin E. P., Sergeyev B. L. (2016). The level of IgG antibodies reactive to TF, Tn and alpha-Gal polyacrylamide-glycoconjugates in breast cancer patients: relation to survival. *Experimental Oncology*.

[35] Heimburg J., Yan J., Morey S. (2006). Inhibition of Spontaneous Breast Cancer Metastasis by Anti—Thomsen- Friedenreich Antigen Monoclonal Antibody JAA-F11. *Neoplasia*.

[36] Almogren A., Abdullah J., Ghapure K., Ferguson K., Glinsky V. V., Rittenhouse-Olson K. (2012). Anti-Thomsen-Friedenreich-Ag (anti-TF-Ag) potential for cancer therapy. *Frontiers in Bioscience*.

[37] Springer G. F., Desai P. R., Tegtmeyer H., Spencer B. D., Scanlon E. F. (1993). Pancarcinoma T/Tn antigen detects human carcinoma long before biopsy does and its vaccine prevents breast carcinoma recurrence. *Annals of the New York Academy of Sciences*.

[38] Springer G. F., Desai P. R., Tegtmeyer H., Carlstedt S. C., Scanlon E. F. (1994). T/Tn antigen vaccine is effective and safe in preventing recurrence of advanced human breast carcinoma. *Cancer Biotherapy*.

[39] Ferguson K., Yadav A., Morey S. (2014). Preclinical studies with JAA-F11 anti-Thomsen-Friedenreich monoclonal antibody for human breast cancer. *Future Oncology*.

[40] Tati S., Fisk J. C., Abdullah J. (2017). Humanization of JAA-F11, a Highly Specific Anti-Thomsen-Friedenreich Pancarcinoma Antibody and In Vitro Efficacy Analysis. *Neoplasia*.

[41] Hakomori S. (2002). Glycosylation defining cancer malignancy: new wine in an old bottle. *Proceedings of the National Academy of Sciences*.

[42] Yu L. G. (2007). The oncofetal Thomsen-Friedenreich carbohydrate antigen in cancer progression. *Glycoconjugate Journal*.

[43] Kailemia M. J., Park D., Lebrilla C. B. (2017). Glycans and glycoproteins as specific biomarkers for cancer. *Analytical and Bioanalytical Chemistry*.

[44] Desai P. R. (2000). Immunoreactive T and Tn antigens in malignancy: role in carcinoma diagnosis, prognosis, and immunotherapy. *Transfusion Medicine Reviews*.

[45] Cao Y., Karsten U. R., Liebrich W., Haensch W., Springer G. F., Schlag P. M. (1995). Expression of Thomsen-Friedenreich-related antigens in primary and metastatic colorectal carcinomas. A reevaluation. *Cancer*.

[46] Barr N., Taylor C. R., Young T., Springer G. F. (1989). Are pancarcinoma T and Tn differentiation antigens?. *Cancer*.

[47] Li F., Glinskii O. V., Mooney B. P., Rittenhouse-Olson K., Pienta K. J., Glinsky V. V. (2017). Cell surface Thomsen-Friedenreich proteome profiling of metastatic prostate cancer cells reveals potential link with cancer stem cell-like phenotype. *Oncotarget*.

[48] Singh R., Campbell B. J., Yu L.-G. (2001). Cell surface-expressed Thomsen-Friedenreich antigen in colon cancer is predominantly carried on high molecular weight splice variants of CD44. *Glycobiology*.

[49] Karsten U., Goletz S. (2015). What controls the expression of the core-1 (Thomsen-Friedenreich) glycotope on tumor cells?. *Biochemistry (Mosc)*.

[50] Bhatia R., Gautam S. K., Cannon A. (2019). Cancer-associated mucins: role in immune modulation and metastasis. *Cancer and Metastasis Reviews*.

[51] Yi B., Zhang M., Schwartz-Albiez R., Cao Y. (2011). Mechanisms of the apoptosis induced by CD176 antibody in human leukemic cells. *International Journal of Oncology*.

[52] Mereiter S., Polom K., Williams C. (2018). The Thomsen-Friedenreich antigen: a highly sensitive and specific predictor of microsatellite instability in gastric cancer. *Journal of Clinical Medicine*.

[53] Lomax-Browne H. J., Robertson C., Antonopoulos A. (2019). Serum IgA1 shows increased levels of *α* 2,6-linked sialic acid in breast cancer. *Interface Focus*.

[54] Karsten U., Goletz S. (2013). What makes cancer stem cell markers different?. *SpringerPlus*.

[55] Lin W. M., Karsten U., Goletz S., Cheng R. C., Cao Y. (2011). Expression of CD176 (Thomsen-Friedenreich antigen) on lung, breast and liver cancer-initiating cells. *International Journal of Experimental Pathology*.

[56] Ulsemer P., Toutounian K., Kressel G. (2016). Impact of oral consumption of heat-treated Bacteroides xylanisolvens DSM 23964 on the level of natural TF*α*-specific antibodies in human adults. *Beneficial Microbes*.

[57] Henderson G., Ulsemer P., Schöber U. (2011). Occurrence of the human tumor-specific antigen structure Gal*β*1-3GalNAc*α*- (Thomsen-Friedenreich) and related structures on gut bacteria: prevalence, immunochemical analysis and structural confirmation. *Glycobiology*.

[58] Klaamas K., Kurtenkov O., Rittenhouse-Olson K. (2002). Expression of tumorassociated Thomsen-Friedenreich antigen (T Ag) in Helicobacter pylori and modulation of T Ag specific immune response in infected individuals. *Immunological Investigations*.

[59] Chang C. J., Chiu N. C., Huang F. Y. (2019). Predictive value of Thomsen-Friedenreich antigen activation for Streptococcus pneumoniae infection and severity in pediatric lobar pneumonia. *Journal of Microbiology, Immunology, and Infection*.

[60] Seger R., Joller P., Baerlocher K., Hitzig W. H. (1980). Neuraminidase-producing pneumococci in the pathogenesis of hemolytic-uremic syndrome. *Schweizerische Medizinische Wochenschrift*.

[61] Moh-Klaren J., Bodivit G., Jugie M. (2017). Severe hemolysis after plasma transfusion in a neonate with necrotizing enterocolitis, Clostridium perfringens infection, and red blood cell T-polyagglutination. *Transfusion*.

[62] Shinozuka T. (1994). Changes in human red blood cells during aging *in vivo*. *The Keio Journal of Medicine*.

[63] Peixoto A., Relvas-Santos M., Azevedo R., Santos L. L., Ferreira J. A. (2019). Protein glycosylation and tumor microenvironment alterations driving cancer hallmarks. *Frontiers in Oncology*.

[64] Zhang Z., Wuhrer M., Holst S. (2018). Serum sialylation changes in cancer. *Glycoconjugate Journal*.

[65] Scott E., Munkley J. (2019). Glycans as biomarkers in prostate cancer. *International Journal of Molecular Sciences*.

[66] Chen J., Fang M., Zhao Y. P. (2015). Serum N-glycans: a new diagnostic biomarker for light chain multiple myeloma. *PLoS One*.

[67] Richter D. U., Jeschke U., Makovitzky J. (2000). Expression of the Thomsen-Friedenreich (TF) antigen in the human placenta. *Anticancer Research*.

[68] Heimburg-Molinaro J., Almogren A., Morey S. (2009). Development, characterization, and immunotherapeutic use of peptide mimics of the Thomsen-Friedenreich carbohydrate antigen. *Neoplasia*.

[69] Kieber-Emmons T., Saha S., Pashov A., Monzavi-Karbassi B., Murali R. (2014). Carbohydrate-mimetic peptides for pan anti-tumor responses. *Frontiers in Immunology*.

[70] Niwa R., Satoh M. (2015). The current status and prospects of antibody engineering for therapeutic use: focus on glycoengineering technology. *Journal of Pharmaceutical Sciences*.

[71] Yu X., Marshall M. J. E., Cragg M. S., Crispin M. (2017). Improving Antibody-Based cancer therapeutics through glycan engineering. *BioDrugs*.

[72] Avrameas S. (1991). Natural autoantibodies: from ‘horror autotoxicus’ to ‘gnothi seauton’. *Immunology Today*.

[73] Butschak G., Karsten U. (2002). Isolation and characterization of Thomsen-Friedenreich-specific antibodies from human serum. *Tumour Biology*.

[74] Schneider C., Smith D. F., Cummings R. D. (2015). The human IgG anti-carbohydrate repertoire exhibits a universal architecture and contains specificity for microbial attachment sites. *Science Translational Medicine*.

[75] Novak J., Julian B. A., Tomana M., Mestecky J. (2008). IgA glycosylation and IgA immune complexes in the pathogenesis of IgA nephropathy. *Seminars in Nephrology*.

[76] Seeling M., Brückner C., Nimmerjahn F. (2017). Differential antibody glycosylation in autoimmunity: sweet biomarker or modulator of disease activity?. *Nature Reviews Rheumatology*.

[77] Grader-Beck T., Boin F., von Gunten S., Smith D., Rosen A., Bochner B. S. (2011). Antibodies recognising sulfated carbohydrates are prevalent in systemic sclerosis and associated with pulmonary vascular disease. *Annals of the Rheumatic Diseases*.

[78] Desai P. R., Ujjainwala L. H., Carlstedt S. C., Springer G. F. (1995). Anti-Thomsen-Friedenreich (T) antibody-based ELISA and its application to human breast carcinoma detection. *Journal of Immunological Methods*.

[79] Bovin N. V. (1998). Polyacrylamide-based glycoconjugates as tools in glycobiology. *Glycoconjugate Journal*.

[80] Smorodin E. P., Kurtenkov O. A., Sergeyev B. L., Klaamas K. V. (2011). The characterization of Cross-reactive antibodies to Thomsen- *Friedenreich a/b* and related glycan-conjugates with polyacrylamide carriers in patients with gastrointestinal cancer. *Journal of Clinical & Cellular Immunology*.

[81] Kurtenkov O., Klaamas K., Miljukhina L. (1995). The lower level of natural anti-Thomsen-Friedenreich antigen (TFA) agglutinins in sera of patients with gastric cancer related to ABO(H) blood-group phenotype. *International Journal of Cancer*.

[82] Smorodin E. P., Kurtenkov O. A., Sergeyev B. L., Kodar K. E., Chuzmarov V. I., Afanasyev V. P. (2008). Postoperative change of anti-Thomsen-Friedenreich and Tn IgG level: the follow-up study of gastrointestinal cancer patients. *World Journal of Gastroenterology*.

[83] Muthana S. M., Gildersleeve J. C. (2016). Factors affecting anti-glycan IgG and IgM repertoires in human serum. *Scientific Reports*.

[84] Ulsemer P., Henderson G., Toutounian K. (2013). Specific humoral immune response to the Thomsen-Friedenreich tumor antigen (CD176) in mice after vaccination with the commensal bacterium Bacteroides ovatus D-6. *Cancer Immunology, Immunotherapy*.

[85] Klaamas K., Kurtenkov O., Brjalin V., Miljukhina L., Shljapnikova L., Engstrand L. (2002). Enhanced humoral immune response to tumor-associated T glycotope (Ga*β*1,3-GalNAc) in Helicobacter pylori-infected blood donors, patients with gastric cancer and benign gastric conditions. *Experimental Oncology*.

[86] Klaamas K., Kurtenkov O., Ellamaa M., Wadström T. (1997). The Helicobacter pylori seroprevalence in blood donors related to Lewis (a,b) histo-blood group phenotype. *European Journal of Gastroenterology & Hepatology*.

[87] Bara J., Imberty A., Pérez S., Imai K., Yachi A., Oriol R. (1993). A fucose residue can mask the MUC-1 epitopes in normal and cancerous gastric mucosae. *International Journal of Cancer*.

[88] Okada Y., Sotozono M., Sakai N., Yonei T., Nakanishi S., Tsuji T. (1994). Fucosylated Thomsen-Friedenreich antigen in alpha-anomeric configuration in human gastric surface epithelia: an allogeneic carbohydrate antigen possibly controlled by the Se gene. *The Journal of Histochemistry and Cytochemistry*.

[89] Kurtenkov O., Klaamas K. (2017). Hidden IgG Antibodies to the Tumor-Associated Thomsen-Friedenreich Antigen in Gastric Cancer Patients: Lectin Reactivity, Avidity, and Clinical Relevance. *BioMed Research International*.

[90] Springer G. F., Desai P. R. (1975). Depression of Thomsen-Friedenreich (anti-T) antibody in humans with breast carcinoma. *Die Naturwissenschaften*.

[91] Springer G. F., Desai P. R. (1975). Increase in anti-T titer scores of breast-carcinoma patients following mastectomy. *Die Naturwissenschaften*.

[92] Kurtenkov O., Innos K., Sergejev B., Klaamas K. (2018). The Thomsen-Friedenreich Antigen-Specific Antibody Signatures in Patients with Breast Cancer. *BioMed Research International*.

[93] Kurtenkov O., Bubina M., Klaamas K. (2018). Signatures of anti-Thomsen- Friedenreich antigen antibody diversity in colon cancer patients. *Experimental Oncology*.

[94] Kurtenkov O., Klaamas K. (2015). Increased avidity of the Sambucus nigra lectin-reactive antibodies to the Thomsen-Friedenreich antigen as a potential biomarker for gastric cancer. *Disease Markers*.

[95] Heimburg-Molinaro J., Lum M., Vijay G., Jain M., Almogren A., Rittenhouse-Olson K. (2011). Cancer vaccines and carbohydrate epitopes. *Vaccine*.

[96] Son H. Y., Apostolopoulos V., Kim C. W. (2016). T/Tn immunotherapy avoiding immune deviation. *International Journal of Immunopathology and Pharmacology*.

[97] Irazoqui F. J., Nores G. A. (2003). Thomsen-Friedenreich disaccharide immunogenicity. *Current Cancer Drug Targets*.

[98] Yu L. G., Jansson B., Fernig D. G. (1997). Stimulation of proliferation in human colon cancer cells by human monoclonal antibodies against the TF antigen (galactose beta1-3 N-acetyl-galactosamine). *International Journal of Cancer*.

[99] Vogtmann E., Goedert J. J. (2016). Epidemiologic studies of the human microbiome and cancer. *British Journal of Cancer*.

[100] Li G., Yu S., Xu J. (2019). The prognostic role of Helicobacter pylori in gastric cancer patients: A meta- analysis. *Clinics and Research in Hepatology and Gastroenterology*.

[101] Nimmerjahn F., Ravetch J. V. (2007). Antibodies, Fc receptors and cancer. *Current Opinion in Immunology*.

[102] Raju T. S. (2008). Terminal sugars of Fc glycans influence antibody effector functions of IgGs. *Current Opinion in Immunology*.

[103] Kaneko Y., Nimmerjahn F., Ravetch J. V. (2006). Anti-inflammatory activity of immunoglobulin G resulting from Fc sialylation. *Science*.

[104] Böhm S., Schwab I., Lux A., Nimmerjahn F. (2012). The role of sialic acid as a modulator of the anti-inflammatory activity of IgG. *Seminars in Immunopathology*.

[105] Bartsch Y. C., Rahmöller J., Mertes M. M. M. (2018). Sialylated autoantigen-reactive IgG antibodies attenuate disease development in autoimmune mouse models of lupus nephritis and rheumatoid arthritis. *Frontiers in Immunology*.

[106] Mehta A. S., Long R. E., Comunale M. A. (2008). Increased levels of galactose-deficient anti-Gal immunoglobulin G in the sera of hepatitis C virus-infected individuals with fibrosis and cirrhosis. *Journal of Virology*.

[107] Klaamas K., Kodar K., Kurtenkov O. (2008). An increased level of the concanavalin A-positive IgG in the serum of patients with gastric cancer as evaluated by a lectin enzyme-linked immunosorbent assay (LELISA). *Neoplasma*.

[108] Shade K.-T., Anthony R. (2013). Antibody glycosylation and inflammation. *Antibodies*.

[109] Ren S., Zhang Z., Xu C. (2016). Distribution of IgG galactosylation as a promising biomarker for cancer screening in multiple cancer types. *Cell Research*.

[110] Zhang D., Chen B., Wang Y. (2016). Disease-specific IgG Fc N-glycosylation as personalized biomarkers to differentiate gastric cancer from benign gastric diseases. *Scientific Reports*.

[111] Gudelj I., Lauc G., Pezer M. (2018). Immunoglobulin G glycosylation in aging and diseases. *Cellular Immunology*.

[112] Goulabchand R., Vincent T., Batteux F., Eliaou J. F., Guilpain P. (2014). Impact of autoantibody glycosylation in autoimmune diseases. *Autoimmunity Reviews*.

[113] Shih H. C., Chang M. C., Chen C. H. (2019). High accuracy differentiating autoimmune pancreatitis from pancreatic ductal adenocarcinoma by immunoglobulin G glycosylation. *Clinical Proteomics*.

[114] Knopf J., Biermann M. H. C., Munoz L. E., Herrmann M. (2016). Antibody glycosylation as a potential biomarker for chronic inflammatory autoimmune diseases. *AIMS Genetics*.

[115] Maverakis E., Kim K., Shimoda M. (2015). Glycans in the immune system and the altered glycan theory of autoimmunity: a critical review. *Journal of Autoimmunity*.

[116] Nguyen T. T. T., Baumgarth N. (2016). Natural IgM and the development of B cell-mediated autoimmune diseases. *Critical Reviews in Immunology*.

[117] Ruhaak L. R., Kim K., Stroble C. (2016). Protein-specific differential glycosylation of immunoglobulins in serum of ovarian cancer patients. *Journal of Proteome Research*.

[118] Kawaguchi-Sakita N., Kaneshiro-Nakagawa K., Kawashima M. (2016). Serum immunoglobulin G Fc region N-glycosylation profiling by matrix-assisted laser desorption/ionization mass spectrometry can distinguish breast cancer patients from cancer-free controls. *Biochemical and Biophysical Research Communications*.

[119] Kodar K., Stadlmann J., Klaamas K., Sergeyev B., Kurtenkov O. (2012). Immunoglobulin G Fc N-glycan profiling in patients with gastric cancer by LC-ESI-MS: relation to tumor progression and survival. *Glycoconjugate Journal*.

[120] Tanaka T., Yoneyama T., Noro D. (2017). Aberrant N-glycosylation profile of serum immunoglobulins is a diagnostic biomarker of urothelial carcinomas. *International Journal of Molecular Sciences*.

[121] Xu P.-C., Gou S.-J., Yang X.-W. (2012). Influence of variable domain glycosylation on anti-neutrophil cytoplasmic autoantibodies and anti-glomerular basement membrane autoantibodies. *BMC Immunology*.

[122] Alter G., Ottenhoff T. H. M., Joosten S. A. (2018). Antibody glycosylation in inflammation, disease and vaccination. *Seminars in Immunology*.

[123] Ohmi Y., Ise W., Harazono A. (2016). Sialylation converts arthritogenic IgG into inhibitors of collagen-induced arthritis. *Nature Communications*.

[124] Ackerman M. E., Crispin M., Yu X. (2013). Natural variation in `n of HIV-specific antibodies impacts antiviral activity. *The Journal of Clinical Investigation*.

[125] Mahan A. E., Jennewein M. F., Suscovich T. (2016). Antigen-specific antibody glycosylation is regulated via vaccination. *PLoS Pathogens*.

[126] Scherer H. U., van der Woude D., Ioan-Facsinay A. (2010). Glycan profiling of anti-citrullinated protein antibodies isolated from human serum and synovial fluid. *Arthritis and Rheumatism*.

[127] Wuhrer M., Selman M. H., McDonnell L. A. (2015). Proinflammatory pattern of IgG1 Fc glycosylation in multiple sclerosis cerebrospinal fluid. *Journal of Neuroinflammation*.

[128] Kodar K., Kurtenkov O., Klaamas K. (2009). The Thomsen-Friedenreich antigen and alphaGal-specific human IgG glycoforms: concanavalin A reactivity and relation to survival of cancer patients. *Immunological Investigations*.

[129] Kurtenkov O., Izotova J., Klaamas K., Sergeyev B. (2014). Increased sialylation of anti-Thomsen-Friedenreich antigen (CD176) antibodies in patients with gastric cancer: a diagnostic and prognostic potential. *BioMed Research International*.

[130] Kurtenkov O., Bubina M., Klaamas K. (2018). Signatures of antiThomsen- Friedenreich antigen antibody diversity in colon cancer patients. *Experimental Oncology*.

[131] Kodar K., Izotova J., Klaamas K., Sergeyev B., Järvekülg L., Kurtenkov O. (2013). Aberrant glycosylation of the anti-Thomsen-Friedenreich glycotope immunoglobulin G in gastric cancer patients. *World Journal of Gastroenterology*.

[132] Gerçel-Taylor C., Bazzett L. B., Taylor D. D. (2001). Presence of aberrant tumor-reactive immunoglobulins in the circulation of patients with ovarian cancer. *Gynecologic Oncology*.

[133] Sindrewicz P., Lian L.-Y., Yu L.-G. (2016). Interaction of the oncofetal Thomsen–Friedenreich antigen with galectins in cancer progression and metastasis. *Frontiers in Oncology*.

[134] Glinsky V. V., Glinsky G. V., Rittenhouse-Olson K. (2001). The role of Thomsen-Friedenreich antigen in adhesion of human breast and prostate cancer cells to the endothelium. *Cancer Research*.

[135] Sharon N. (2006). Carbohydrates as future anti-adhesion drugs for infectious diseases. *Biochimica et Biophysica Acta*.

[136] Yu L. G., Andrews N., Zhao Q. (2007). Galectin-3 interaction with Thomsen-Friedenreich disaccharide on cancer-associated MUC1 causes increased cancer cell endothelial adhesion. *Journal of Biological Chemistry*.

[137] Wolf M. F., Ludwig A., Fritz P., Schumacher K. (1988). Increased expression of Thomsen-Friedenreich antigens during tumor progression in breast cancer patients. *Tumour Biology*.

[138] Nakamori S., Ota D. M., Cleary K. R., Shirotani K., Irimura T. (1994). MUC1 mucin expression as a marker of progression and metastasis of human colorectal carcinoma. *Gastroenterology*.

[139] Baldus S. E., Hanisch F. G., Monaca E. (1999). Immunoreactivity of Thomsen-Friedenreich (TF) antigen in human neoplasms: the importance of carrier-specific glycotope expression on MUC1. *Histology and Histopathology*.

[140] Karsten U., von Mensdorff-Pouilly S., Goletz S. (2005). What makes MUC1 a tumor antigen?. *Tumour Biology*.

[141] Baldus S. E., Wienand J. R., Werner J. P. (2005). Expression of MUC1, MUC2 and oligosaccharide epitopes in breast cancer: prognostic significance of a sialylated MUC1 epitope. *International Journal of Oncology*.

[142] Iurisci I., Tinari N., Natoli C., Angelucci D., Cianchetti E., Iacobelli S. (2000). Concentrations of galectin-3 in the sera of normal controls and cancer patients. *Clinical Cancer Research*.

[143] Zhao Q., Guo X., Nash G. B. (2009). Circulating galectin-3 promotes metastasis by modifying MUC1 localization on cancer cell surface. *Cancer Research*.

[144] Liu J., Yi B., Zhang Z., Cao Y. (2016). CD176 single-chain variable antibody fragment inhibits the adhesion of cancer cells to endothelial cells and hepatocytes. *Frontiers in Medicine*.

[145] Santarsia S., Grosso A. S., Trovão F. (2018). Molecular recognition of a Thomsen-Friedenreich antigen mimetic targeting human galectin-3. *ChemMedChem*.

[146] Chung A. W., Crispin M., Pritchard L. (2014). Identification of antibody glycosylation structures that predict monoclonal antibody Fc-effector function. *AIDS*.

[147] Li T., DiLillo D. J., Bournazos S., Giddens J. P., Ravetch J. V., Wang L.-X. (2017). Modulating IgG effector function by Fc glycan engineering. *Proceedings of the National Academy of Sciences*.

[148] Vesely M. D., Kershaw M. H., Schreiber R. D., Smyth M. J. (2011). Natural innate and adaptive immunity to cancer. *Annual Review of Immunology*.

[149] Irazoqui F. J., Jansson B., Lopez P. H. H., Nores G. A. (2001). Correlative Fine specificity of several Thomsen-Friedenreich disaccharide-binding proteins with an effect on Tumor cell proliferation. *Journal of Biochemistry*.

